# Testing mapping algorithms of the cancer-specific EORTC QLQ-C30 onto EQ-5D in malignant mesothelioma

**DOI:** 10.1186/s12955-014-0196-y

**Published:** 2015-01-23

**Authors:** David T Arnold, Donna Rowen, Matthijs M Versteegh, Anna Morley, Clare E Hooper, Nicholas A Maskell

**Affiliations:** Academic Respiratory Unit, School of Clinical Sciences, University of Bristol, Bristol, UK; School of Health and Related Research (ScHARR), University of Sheffield, Sheffield, UK; Institute for Medical Technology Assessment, Erasmus University of Rotterdam, Rotterdam, Netherlands

**Keywords:** EQ-5D, QLQ-C30, Mapping, Mesothelioma, Health technology assessment, QALY

## Abstract

**Background:**

In order to estimate utilities for cancer studies where the EQ-5D was not used, the EORTC QLQ-C30 can be used to estimate EQ-5D using existing mapping algorithms. Several mapping algorithms exist for this transformation, however, algorithms tend to lose accuracy in patients in poor health states. The aim of this study was to test all existing mapping algorithms of QLQ-C30 onto EQ-5D, in a dataset of patients with malignant pleural mesothelioma, an invariably fatal malignancy where no previous mapping estimation has been published.

**Methods:**

Health related quality of life (HRQoL) data where both the EQ-5D and QLQ-C30 were used simultaneously was obtained from the UK-based prospective observational SWAMP (South West Area Mesothelioma and Pemetrexed) trial. In the original trial 73 patients with pleural mesothelioma were offered palliative chemotherapy and their HRQoL was assessed across five time points. This data was used to test the nine available mapping algorithms found in the literature, comparing predicted against observed EQ-5D values. The ability of algorithms to predict the mean, minimise error and detect clinically significant differences was assessed.

**Results:**

The dataset had a total of 250 observations across 5 timepoints. The linear regression mapping algorithms tested generally performed poorly, over-estimating the predicted compared to observed EQ-5D values, especially when observed EQ-5D was below 0.5. The best performing algorithm used a response mapping method and predicted the mean EQ-5D with accuracy with an average root mean squared error of 0.17 (Standard Deviation; 0.22). This algorithm reliably discriminated between clinically distinct subgroups seen in the primary dataset.

**Conclusions:**

This study tested mapping algorithms in a population with poor health states, where they have been previously shown to perform poorly. Further research into EQ-5D estimation should be directed at response mapping methods given its superior performance in this study.

**Electronic supplementary material:**

The online version of this article (doi:10.1186/s12955-014-0196-y) contains supplementary material, which is available to authorized users.

## Background

With the increasing availability of novel but expensive therapies for cancer, the accuracy of health technology assessment becomes ever more important. The National Institute for Health and Care Excellence (NICE) favours the use of Quality Adjusted Life Years (QALYs) for such analyses [1], which combine life years with a measure of health-related quality of life (HRQoL), called utilities [2]. Utility values are measured on a 1 to 0 (full health- dead) scale and are usually obtained using existing off-the-shelf preference based measures, such as the EQ-5D [3], SF-6D [4] or HUI [5] instruments. General population preference derived tariffs are used to convert the health states into utilities. General population values are typically used as the measure will often be used to inform allocation decisions of public funds [6]. Utility values derived from these methods can then be directly multiplied by life years in each health state to generate QALYs.

However, the majority of clinical trials do not use ‘preference based’ measures opting instead for disease specific measure that are not preference-based so cannot be used to estimate QALYs directly. These are deemed more sensitive to subtle treatment benefits, as they contain more varied and disease specific dimensions [7]. In an attempt to reduce patient burden ‘preference based’ measures are often not used [8]. However, agencies such as NICE require the use of preference-based measures to estimate utility values for use in health technology assessment, and NICE in particular recommend the use of EQ-5D [1].

If a trial has not included a preference-based measure such as the EQ-5D, but EQ-5D utilities are required for health technology assessment, two options are available; [1] take approximate utility values from other similar patient datasets available in the literature [2,9-11] use a conversion algorithm to ‘map’ the results of the disease specific measures onto the preference based measures. Method 1 requires existing datasets containing EQ-5D in the patient population, which may not always be available**.** Method 2 uses mapping, a relatively novel technique with the majority of papers on the topic being published in the last decade and only mapping between the most frequently used measures [12].

This study focuses on published mapping algorithms mapping between the most commonly used cancer specific quality of life measure [13], the EORTC QLQ-C30 (hence forth the QLQ C30) and the EQ-5D. The majority of published algorithms use linear regression by ordinary least squares to estimate the relationship between the QLQ-C30 and EQ-5D where both measures have been used on the same patient population (an estimation dataset). The resultant algorithm is then tested, either on small samples of the original population (a technique known as bootstrapping), or ideally onto a different patient population (a validation sample). The performance of the mapping algorithm is assessed on its ability to accurately predict the observed mean EQ-5D value, minimise the mean absolute error where the error is generated using the difference between predicted and observed EQ-5D values, both for the entire sample and across the severity range [14]. It is also of importance that the mapped EQ-5D values can also detect clinically important HRQoL differences between subgroups of the population that were observed using EQ-5D in the original study. It is of note that no guidelines exist for acceptable mapping performance [15]. Several studies have been published which attempt to map the QLQ-C30 onto EQ-5D and have been developed from several different patient populations including breast, gastric, haematological and lung cancer. All have been shown to perform well within their validation samples taken from the same patient populations. However, performance of algorithms tends to worsen in patients with poor health states, with over-prediction of predicted against observed values [16]. A recent Health Technology Assessment monograph published by Longworth et al. supported the use of a response mapping technique where logistic regression is used to fit models to the same dimensions of the EQ-5D as opposed to the index score [17]. The algorithm generated performed well against its validation dataset, but has not been tested on external datasets or compared to linear regression mapping algorithms.

It is recommended that users should select an algorithm estimated using a sample with similar characteristics to the patient dataset they are applying the algorithm to [14]. However there is little guidance regarding the choice of algorithm where patient characteristics differ between the estimation and application dataset both in terms of the condition and hence dimensions of health which are likely to be important, and in terms of the severity range of the dataset. This study aims to test the existing mapping algorithms for QLQ-C30 onto the EQ-5D using a dataset of patients with inoperable pleural mesothelioma, a patient group with particularly poor health states where minimal published EQ-5D literature currently exists and mapping has never been attempted. It is in such populations where mapping accuracy is more uncertain and arguably more important, as therapies may only have modest life year benefits and a focus on HRQoL.

## Methods

### Population

The HRQoL data for this study was drawn from the SWAMP (South West Area Mesothelioma and Pemetrexed) trial. This UK based multicentre observational study recruited 73 patients with newly diagnosed malignant pleural mesothelioma deemed unsuitable for surgery by the multi-disciplinary team. Patients had to have treatment naïve mesothelioma and be in WHO performance score 0, 1 or 2. Patients were excluded if considered too unwell for chemotherapy or had a life expectancy of less than 3 months. Those recruited were offered chemotherapy with pemetrexed and cisplatin (the standard chemotherapy regimen in the UK) after written informed consent. Pleural mesothelioma is a cancer of the pleural lining of the lung. It has been directly correlated with occupational exposure to asbestos, and for this reason predominates in males [18]. Malignant mesothelioma is a rapidly progressive cancer with respect to symptomatology with patients suffering from worsening shortness of breath (dyspnoea), night sweats, fatigue and chest pain, all of which have a significant impact on HRQoL. In general survival is poor, with a median survival of 7 to 11 months after diagnosis [19].

Health-related quality of life (HRQoL) of these 73 patients was assessed at 5 different time-points (baseline, 6 weeks, 16 weeks, 9 months and 12 months). The patients completed the questionnaires for the first 2 time-points (at baseline and 6 weeks) under the direct supervision of either the trial co-ordinator or the trial research nurse so that they could go through the questions and answer any queries they had on how to complete. The other questionnaires were posted to the patients to complete at home and returned in an enclosed stamped addressed envelope and advised them to contact a member of the research team if they were unsure of how to answer any of the questions. Four different HRQoL measures were used at each time point, namely the EQ-5D, EORTC QLQ-C30, EORTC LC13 and the Edmonton Symptom Assessment Score. This study focuses on the comparison between EQ-5D and QLQ-C30. Due to the rapid progression of this condition there was significant loss of comparisons at later time points, due to patient death or being too unwell to complete the questionnaires, with a total number of direct comparisons of 250. See Additional file 1 for full details on missing data by timepoints.

### Ethical approval and registration

Ethical and regulatory approval for the primary study was obtained before recruitment commenced (UK REC Reference: 08/H0102/46). The trial was registered in the national portfolio (UKCRN ID: 8450).

### Instruments

The EQ-5D is a widely used preference based generic HRQoL instrument. It measures health-related quality of life on 5 dimensions (mobility, self-care, usual activities, pain/discomfort, and anxiety/depression) with 3 severity levels for each dimension, producing 243 health states in total. A utility score can be generated for each health state by applying country specific general population-elicited tariffs, which can then be used to calculate QALYs. This dataset used the UK population EQ-5D tariff [3].

The QLQ-C30 (version 3) is a disease specific questionnaire developed specifically for use in cancer. There are 5 functional scales (physical, role, emotional, cognitive and social), a global health item, 3 symptom scales (fatigue, nausea/vomiting and pain), and single symptom items (dyspnoea, appetite loss, constipation, diarrhoea and financial difficulties). All items are converted onto a 0 to 100 scale. It is important to note that higher scores for the global health and functional categories indicate higher functioning, whereas higher symptom scores denote worse symptomatology. With a total of 30 questions the instrument gives a broad and sensitive assessment of HRQoL in patients with cancer [13]. Preference-based measures have been derived from the QLQ-C30 enabling the QLQ-C30 to be directly used to estimate QALYs [20,21]. However, some agencies, and NICE in particular, recommend utility values generated using a generic preference-based measure and for the purposes of comparability across health technology assessment undertaken across different conditions and patient populations recommend the use of EQ-5D in particular [14].

### Mapping algorithms

A literature search of Pubmed and a recently constructed database of mapping algorithms [12] identified 9 studies that had attempted to map the QLQ-C30 onto the EQ-5D [8,22-28]. Table 1 summarises the mapping algorithms found and the estimation populations they were derived from, as well as the validation population used to test the resultant algorithm. The full algorithms can be found in the Additional file 1. Also included are the county specific tariffs used in the calculation of the original EQ-5D values. The tariff used has a major impact on the EQ-5D values produced. The UK tariff for example will generate significantly lower utility values than the US alternative for the same questionnaire responses in the order of 0.05 to 0.23 [29-31]. For this reason this study has generated EQ-5D values using the UK, US, Korean and Dutch tariffs, so the mapping algorithms are tested against their country specific EQ-5D values.

**Table 1:** **Summary of mapping algorithms**

**Mapping algorithm primary author**	**Estimation Population (number of comparisons)**	**Validation Population (number of comparisons)**	**Mean EQ-5D (Country Specific Tariff used)**	**Regression method**	**No. of model variables**	**Mean QLQ-C30 Global Health**
Crott [22]	RCT of breast cancer therapies (n = 798)	Bootstrapping	0.76 (UK)	OLS	12	64.4
Jang [8]	Consecutive patients attending an outpatient clinic with non-small cell lung cancer (n = 172)	Bootstrapping	0.76 (US)	Linear regression	15	65.9
Kim EJ [23]	Cross sectional survey of patients with metastatic breast cancer receiving palliative chemotherapy (n = 149)	Breast cancer patients not used in the estimation sample (n = 50)	0.67 (Korean)	OLS	5	53.3
Kim SH [24]	Cross sectional study of patients with different types of cancer receiving chemotherapy (n = 893)	Colon cancer patients (n = 123)	0.82 (Korean)	OLS	5	59.8
Kontodimopoulos [25]	Cross sectional survey of patients with gastric cancer receiving chemotherapy (n = 48)	Bootstrapping	0.55 (UK)	OLS	3	46.4
Longworth [17]	Patients from 3 studies with breast, lung and haematological cancer (n = 771)	n/a	0.58 (UK)	Response mapping	14 plus age and gender	53.0
McKenzie [26]	RCT of palliative therapies for patients with inoperable oesophageal cancer (n = 877)	Low risk breast cancer patients receiving radiotherapy (n = 991)	0.54 (UK)	OLS	15	45.3
Proskorovsky [27]	Cohort study of patients with multiple myeloma (n = 154)	Bootstrapping	0.73 (UK)	Linear regression	4	60.1
Versteegh [28]	Patients with multiple myeloma in an RCT of treatment (n = 723)	Patients with non-Hodgkins lymphoma (n = 789)	0.74 (Dutch)	OLS	11	68.7

OLS- Ordinary Least Squares, RCT- Randomised Controlled Trial.

### Statistical analysis

Several statistical methods were used to assess the ability of the algorithms to map the QLQ-C30 to the EQ-5D. Firstly, the raw differences between observed and predicted mean EQ-5D for the entire dataset (with all 250 comparisons pooled) and across sub-groups were calculated to give an indication as to overall algorithm performance across the dataset. For the pooled data (n-250) a paired T-test was carried out to comparing the observed versus predicted EQ-5D means (p < ·05). A significant result indicates that the mapping has not accurately predicted the observed mean. To give an indication as to the spread about the mean generated by the algorithms, mean absolute error (MAE) and root mean squared error (RMSE) figures were generated. MAE is generated using the difference between observed and predicted EQ-5D at the observational level. RMSE is more sensitive to extreme deviations from the mean and is calculated by root of the average of the squared differences between observed and predicted EQ-5D. Smaller values of MAE and RMSE indicate better algorithm performance.

Given that previous literature has shown that the algorithms perform less well in poor health states the entire dataset was divided into 3 groups depending on observed EQ-5D value (UK tariff) and the above statistical tests repeated. Functional cut-offs were selected to give good distribution of comparisons between the 3 groups, namely observed EQ-5D 1.0 to 0.75, 0.75 to 0.5, and less than 0.5.

The ability of the algorithms to differentiate between clinical groups was assessed. For this we selected 3 significant HRQoL findings seen in the primary study (see below), which used an analysis of co-variance (ANCOVA) test from baseline EQ-5D to 16 weeks (n.b. the same results are seen regardless of the country specific EQ-5D tariff used). The same analysis (ANCOVA) was run using the predicted EQ-5D and results compared to the primary study findings;

1. Patients who received palliative chemotherapy had significantly better HRQoL at 16 weeks than those that did not (ANCOVA p = ·005).
2. Patients with epithelial mesothelioma had significantly better HRQoL at 16 weeks compared to the more aggressive sarcomatoid or biphasic subtypes (ANCOVA p = ·006).
3. Patients with a falling mesothelin (a novel biomarker for mesothelioma severity) had better HRQoL at 16 weeks compared to those with a rising mesothelin (ANCOVA p = ·002).

Finally, to investigate the best performing algorithms ability to correctly predict the observed distribution of EQ-5D values a Kolmogorov-Smirnov test of equality of distribution was used. A statistically significant result (P < ·05) indicates that the predicted distribution is significantly different to the observed distribution seen in the original study.

## Results

After post-hoc assessment of the following analyses the best performing algorithm was the response-mapping Longworth algorithm. Table 2 presents a summary of the mesothelioma dataset used to test the mapping algorithms. The population on average have poor health states with specific difficulties in areas expected with advanced mesothelioma, namely physical functioning and dyspnoea. The population is predominately male (86%), with a mean age of 70 (range 49 to 89). There were 19 (26%), 48 (66%) and 6 (8%) patients in WHO performance classes 0, 1 and 2 respectively. The median survival was 406 days from diagnosis (range 67 to 1734 days).

**Table 2:** **Dataset summary**

**Dimension**	**Mean (SD)**	**Dataset range (Dimension range)**
EQ-5D Utility UK Tariff	0.657 (0.253)	−0.135-1.0 (−0.594-1.0)
EQ-5D Utility US Tariff	0.743 (0.174)	0.145-1.0 (−0.109-1.0)
EQ-5D Utility Dutch Tariff	0.711 (0.223)	−0.050-1.0 (−0.329-1.0)
EQ-5D Utility Korean Tariff	0.735 (0.159)	0.100-1.0 (−0.171-1.0)
EORTC QLQ C-30		
Global Health Status	56.3 (23.3)	0-100 (0–100)
Physical Functioning	65.4 (23.2)	0-100 (0–100)
Role Functioning	54.5 (31.3)	0-100 (0–100)
Emotional Functioning	79.3 (23.8)	0-100 (0–100)
Cognitive Functioning	76.4 (25.8)	0-100 (0–100)
Social Function	63.4 (32.5)	0-100 (0–100)
Fatigue	45.9 (26.6)	0-100 (0–100)
Nausea/Vomiting	12.5 (18.3)	0-100 (0–100)
Pain	27.4 (27.4)	0-100 (0–100)
Dyspnoea	43.7 (28.0)	0-100 (0–100)
Insomnia	30.7 (32.9)	0-100 (0–100)
Appetite	28.4 (31.3)	0-100 (0–100)
Constipation	21.6 (29.0)	0-100 (0–100)
Diarrhoea	7.4 (18.8)	0-100 (0–100)
Financial Problems	10.1 (22.1)	0-100 (0–100)

The results of the 9 mapping algorithms tested across all pooled comparisons (n = 250) are shown in Table 3. Of the OLS algorithms the McKenzie algorithm predicts a group mean value closest to our observed mean, but is not the best performing with respect to root mean squared error (RMSE), as the Jang model has the lowest RMSE. Overall the Longworth response mapping algorithm predicts the mean most accurately with lowest mean absolute error (MAE) and low RMSE. Apart from the Longworth algorithm (p = ∙192), all the mapping algorithms tested had significantly different predicted versus observed EQ-5D means (using a paired T-test at the p < ∙05 level).

**Table 3:** **Summary of mapping performance**

**Mapping algorithm**	**Mean predicted EQ-5D (Observed EQ-5D mean)**	**Predicted EQ-5D mean minus observed mean. (Paired T-test p-value)**	**MAE between predicted and observed values. (SD)**	**Root mean squared error (SD)**
Crott	0.7039 (0.6572)	+0.0467 (<∙001)	0.1316 (0.116)	0.1749 (0.230)
Jang	0.7077 (0.7434)	−0.0357 (<∙001)	0.0351 (0.116)	0.1211 (0.148)
Kim EJ	0.8498 (0.7354)	+0.1144 (<∙001)	0.1144 (0.101)	0.1527 (0.173)
Kim SH	0.8010 (0.7354)	+0.0656 (<∙001)	0.0656 (0.098)	0.1174 (0.149)
Kontodimopoulos	0.7066 (0.6572)	+0.0494 (<∙001)	0.1574 (0.139)	0.2095 (0.281)
Longworth	0.6425 (0.6572)	−0.0147 (∙192)	0.0138 (0.166)	0.1661 (0.216)
McKenzie	0.6294 (0.6572)	−0.0278 (∙023)	0.1439 (0.119)	0.1863 (0.241)
Proskorovsky	0.6032 (0.6572)	−0.0540 (<∙001)	0.0541 (0.180)	0.1865 (0.217)
Versteegh	0.7641 (0.7114)	+0.0527 (<∙001)	0.0528 (0.184)	0.1906 (0.287)

Further analysis was undertaken to assess the performance of the mapping algorithms in different ranges of our dataset. We chose 3 arbitrary cut offs of observed EQ-5D levels (UK tariff), see Table 4.

**Table 4:** **Performance of mapping algorithms in different ranges of observed EQ-5D**

**Mapping algorithm**	**Observed UK EQ-5D values of 0.75-1.00 used (n = 91)**			**Observed UK EQ-5D values of 0.50-0.75 used (n = 114)**			**Observed UK EQ-5D values of less than 0.50 used (n = 45)**		
	**Mean predicted EQ-5D (observed)**	**MAE**	**RMSE**	**Mean predicted EQ-5D (observed)**	**MAE**	**RMSE**	**Mean predicted EQ-5D (observed)**	**MAE**	**RMSE**
Crott	0.8433 (0.8750)	0.0851	0.1086	0.6873 (0.6615)	0.1159	0.1457	0.4639 (0.2064)	0.2656	0.3040
Jang	0.8316 (0.8909)	0.0608	0.1033	0.6773 (0.7410)	0.0637	0.1230	0.5397 (0.4518)	0.0879	0.1462
Kim EJ	0.9430 (0.8826)	0.0604	0.0994	0.8219 (0.7111)	0.1108	0.1343	0.7321 (0.4991)	0.2330	0.2527
Kim SH	0.8937 (0.8826)	0.0111	0.0767	0.7758 (0.7111)	0.0646	0.0968	0.6774 (0.4991)	0.1783	0.2031
Kontodimopoulos	0.9145 (0.8750)	0.1225	0.1569	0.6532 (0.6615)	0.1414	0.1854	0.4215 (0.2064)	0.2684	0.3271
Longworth	0.8105 (0.8750)	0.0667	0.1196	0.6176 (0.6615)	0.0439	0.1556	0.3734 (0.2064)	0.1671	0.2496
McKenzie	0.8206 (0.8750)	0.1180	0.1559	0.5954 (0.6615)	0.1496	0.1937	0.3377 (0.2064)	0.1804	0.2200
Proskorovsky	0.7481 (0.8750)	0.1268	0.1622	0.5596 (0.6615)	0.1019	0.1673	0.4206 (0.2064)	0.2143	0.2634
Versteegh	0.8967 (0.8978)	0.0010	0.0893	0.7387 (0.7134)	0.0255	0.1473	0.5579 (0.3296)	0.2282	0.3598

No one model shows superiority when tested across the dataset, but all perform worse in poor health states, with over-prediction of poor health states and larger errors about the mean. The Jang, Longworth and McKenzie models map most accurately at observed EQ-5D values of less than 0.5 but still suffer from significant over-prediction to a degree of 0.09, 0.17 and 0.13 respectively. The next analysis tests the ability of the mapping algorithms to detect clinical changes seen in observed EQ-5D values of the original dataset.

All 8 linear regression mapping algorithms failed to differentiate between 2 of the 3 clinical subgroups seen in the original study. Only the reduction in HRQoL values seen in the more aggressive cancer subtypes was detected and only the McKenzie model shows a statistically significant relationship. However, the Longworth algorithm selected out all 3 clinical subgroups and was statistically significant in 2 (at the < ∙05 level). See Additional file 2 for full results.

**Figure 1:**
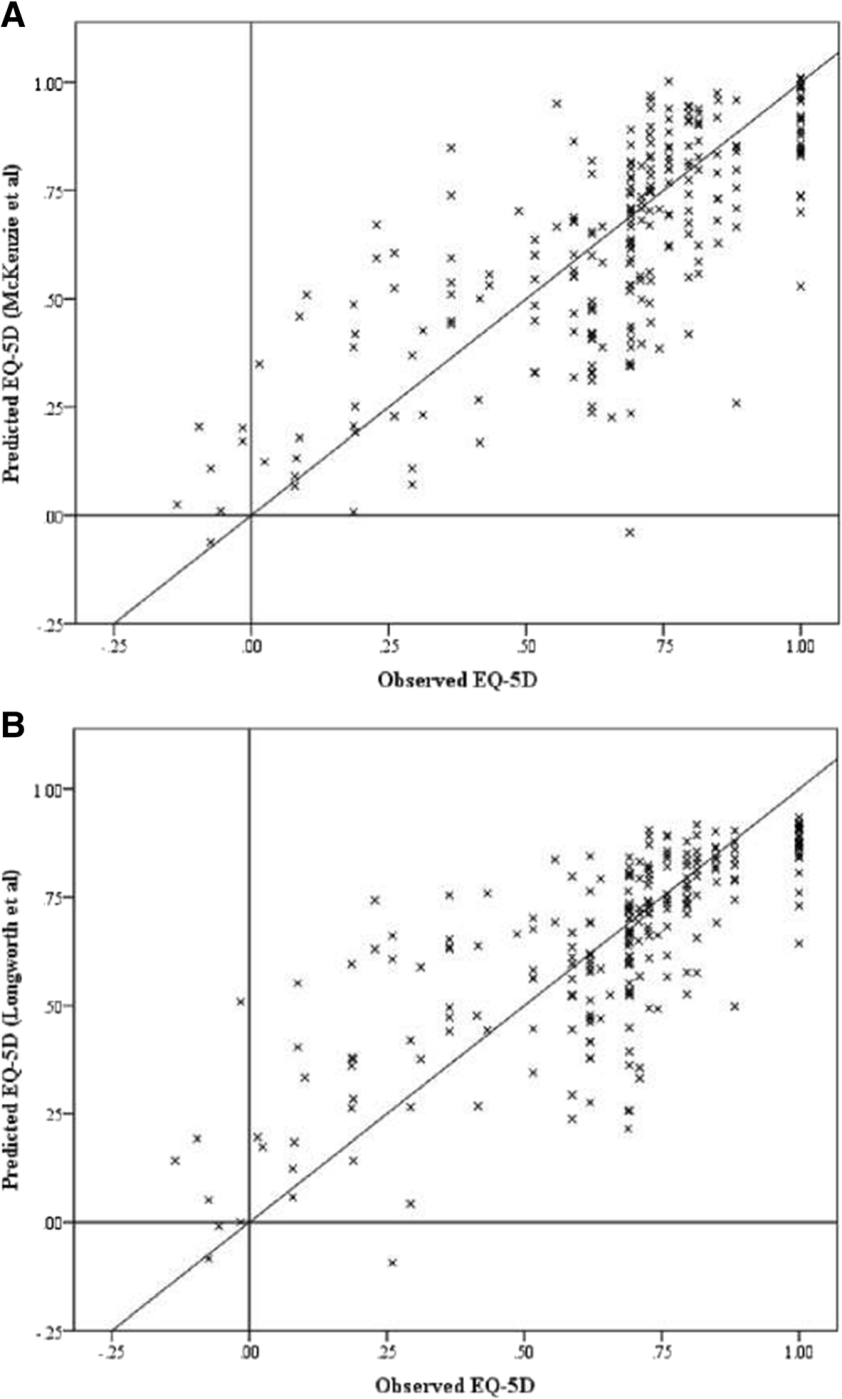
**Scatter plots of observed against predicted values (A) Longworth (B) McKenzie.** (n.b. diagonal line represents x = y, i.e. the result of perfect mapping).

An equality of distributions test (Kolmogorov-Smirnov test) was used to test assess the variability in distributions between the two best performing algorithms and observed EQ-5D values. Both had significantly different distributions (at the p < ·05 level) compared to observed EQ-5D distribution, although the McKenzie algorithm was a strongly significant (p < ·001) and the Longworth was less marked (p = ·026). Visual representations of this data can be found in the Additional file 2. The graphs of the Longworth and McKenzie predicted EQ-5D values against observed values are shown in Figure 1, the lines represent the result of perfect mapping (i.e. x = y). Both algorithms suffer from under-prediction in perfect health, but the Longworth algorithm appears to have less spread about the mean in the intermediate ranges (0.75 up to 1.0). Both algorithms have large over-prediction errors when the observed EQ-5D is less than 0.5.

## Discussion

This study analysed the performance of existing mapping algorithms for converting QLQ C30 scores onto the EQ-5D, a technique required to calculate QALYs for use in health technology assessment. The mapping algorithms were tested on a population with inoperable mesothelioma who had, on average, poor health states (a mean EQ-5D score of 0.66) with 18% of observations with an EQ-5D score below 0.5 using the UK tariff. Based on previous literature [16], it was hypothesised that the mapping algorithms would have large errors in predictions in this dataset with many patients in poor health.

The best 3 performing algorithms across all of the performance criteria were the Jang, McKenzie and Longworth models. The Jang model performed particularly well according to MAE and RMSE across the severity range of the EQ-5D, but was only able to detect 1 of the 3 clinical changes and this was not significant. The McKenzie algorithm overall performed well using all criteria, but was not particularly strong for any one of the criteria. The Longworth algorithm had the smallest difference between observed and predicted mean EQ-5D score, smallest MAE, and was the only algorithm to detect all 3 of the clinical difference. Taking into account all criteria, the Longworth model was selected as the best performing model. It is recognized that this may be considered arbitrary as there is no accepted criteria in the literature for choosing the best performing model and research in this area is encouraged.

The results seen have two elements. First, in patients with ‘perfect’ or ‘near perfect’ health (i.e. values close to 1) the mapping algorithms tended to under-predict the true health states. This is likely a result of the ceiling effect secondary to being close to the upper limit of the scale. This was observed in all 9 mapping algorithms tested and can be seen in Figure 1. That said, this effect was small and had little effect on the overall mean. Second, in patients with poor health all the mapping algorithms over-predicted compared to the observed EQ-5D values. This effect is far greater in some algorithms than others. The algorithms published by Jang, Longworth and McKenzie map relatively well in the subpopulation with very poor health (observed EQ-5D less than 0.5) with a deviation from observed mean of 0.09, 0.17 and 0.13 respectively. In clinical practice this is still a large deviation, but significantly smaller than those seen in the Crott, Kim EJ and Versteegh algorithms of 0.26, 0.23 and 0.23 respectively. Given that the test dataset is in generally poor health the algorithms that vastly over-predicted in the above subgroup also over-predicted the overall mean EQ-5D.

The issue of over-prediction in poor health states is not a novel finding and impacts on health technology assessment when comparisons are made across studies using mapped utility data and studies that directly assessed utilities. It has been noted in the majority of mapping studies, but rarely to the extent seen in this analysis. Other analyses have found its impact is minimal when looking at differences over time or between groups [32] but our results conflict with this conclusion. Its causation is likely to be multifactorial. Firstly, the populations from which these mapping algorithms are derived tend to have better health utilities, so are not designed to cope with poor health states. This might explain why the Longworth and McKenzie algorithms performed better overall, with mean EQ-5D values in their estimation population of 0.58 and 0.54 respectively. Secondly, algorithms are constructed based on a heterogeneous disease population which may have specific characteristics that impact on the mapping algorithm. For example, dyspnoea is an important symptom for the mesothelioma patient population. Six of the linear regression mapping algorithms tested had excluded dyspnoea, and in the other 2 algorithms dyspnoea had a positive (rather than negative, where increased dyspnoea reduces utility) impact on EQ-5D (Kim EJ and McKenzie). In this patient group with mesothelioma, of whom 44% reported some dyspnoea, this could be significantly contributing to overestimation, whereas a mapping algorithm estimated from this patient population would likely include dyspnoea as a negative variable in its algorithm. It has been recommended that when mapping is used, the estimation population should be as clinically similar to the target population as possible to minimise this effect [14]. The ability of the Jang algorithm to limit error about the mean could be attributed to having a similar estimation population (patients with non-small cell lung cancer) to our mesothelioma dataset. Finally the general assumptions of a linear regression model may be suitable for predicting a population mean but inadequate for discriminating between severity levels or clinical subgroups. The superior performance of a response mapping technique is exciting given the limited research in this area to date.

No guidelines exist providing thresholds for acceptable model performance with respect to ability to predict the mean or error about the mean [14]. The studies that produce the algorithms validate them on a validation population or samples of the original population (bootstrapping), and judge accuracy as differences in means of predicted vs. actual values. Across the 9 studies the differences in means in their validation samples ranged from 0 [8] to 0.088 [23], compared to the range we found of 0.028 using the McKenzie to 0.114 with the Kim EJ algorithm. Also, the root mean squared errors (RMSE) in these validation samples are usually reported, ranging from 0.094 [22] to 0.192 [25], compared to our RMSE ranging from 0.121 in the Jang algorithm to 0.300 with the Kontodimopoulos algorithm. Based on these criteria, it is apparent that the mapping studies had difficulty mapping our dataset compared to their original validation studies.

The McKenzie algorithm was the best performing linear regression mapping algorithm tested in terms of predicting the entire dataset’s mean EQ-5D value, and this has been found elsewhere [33]. The algorithm itself was based on a population undergoing palliative therapies for inoperable oesophageal cancer and was generally a population in exceptionally poor health [34]. The average EQ-5D of this population was 0.54 (the lowest of all mapping algorithms tested) with a significant number of patients reporting severe problems within the 5 EQ-5D dimensions. An impressive sample of 877 observations were used to generate the algorithm, and the validation dataset was large (991 observations from 254 patients) and clinically distinct from the estimation sample (trial of radiotherapy for breast cancer in low risk elderly women [35]). They used an ordinary least squares regression to map onto the EQ-5D using all categories of the QLQ-C30. However, despite its robust design there are still concerns regarding its performance. The McKenzie algorithm accurately predicts the mean group EQ-5D, but this may be due to its substantial under prediction in good health states (it performs worst in observed EQ-5D values of greater than 0.75) which may offset the over prediction which still occurs in the poorer health states. The algorithm generated considerable error about the mean which may explain its inability to distinguish between clinically different groups seen in our original study and actually showed the opposite finding in 2 of 3 tested relationships. Our results indicate that response mapping algorithm from Longworth et al. is the most accurate method of distinguishing between clinical subgroups even maintaining statistically significance in 2 of the 3 categories. It showed ability in predicting a population mean and distribution, with reliability in poor health states. It uses a relatively novel technique of response mapping, where QLQ-C30 dimensions are mapped onto specific dimensions of the EQ-5D with the addition of age and gender into the algorithm. The model was constructed from 771 patients with a variety of cancer types (breast- 100, lung- 99, myeloma- 572) and in generally poor health. To our knowledge this is the first study to critically analyse this method outside its estimation dataset.

This study had several limitations which may affect the generalisability and reliability of our results. The analyses were conducted using a dataset with only 250 observations. For this reason we did not attempt to construct a mapping algorithm of our own. However, as datasets of this size are used to generate QALYs, the mapping algorithms should perform accurately, and indeed mapping analysis on pragmatically smaller datasets was encouraged in a recent review [14]. Our population is male dominant (86% male), given the relationship of mesothelioma with asbestos exposure, which could have affected the functionality of the female predominant mapping datasets i.e. breast cancer populations (Kim EJ and Crott). As gender is not included in the EQ-5D or QLQ-C30 scoring systems the effect is likely to be minimal but could further explain why the Longworth algorithm performed well, as it incorporates gender. Although several versions of the EORTC QLQ-C30 exist, we have just based our results on version 3. Although functional levels do vary slightly, the differences are small and excluding studies using other versions would have significantly limited our analysis. Finally we have tested the mapping algorithms that predict different country tariffs of the EQ-5D and tested these in comparison to the relevant country tariff that they aimed to predict. However it is unlikely that a user would be able to choose which country tariff to apply, as typically the analyses would be undertaken for a specific country.

## Conclusions

In summary, a recently published algorithm by Longworth et al. was the best performing of 9 mapping algorithms tested in this poor health population with mesothelioma. It accurately predicted the EQ-5D population mean from QLQ-C30 values with small MAE and RSME. Unlike conventionally constructed algorithms this response mapping technique could discriminate between clinically relevant subgroups. Mapping is always a second best solution to direct collection of EQ-5D values but further research should be directed at response mapping for conversion of disease specific to preference-based measures.

## Additional files

Additional file 1: Table S1. Table of missing questionnaires by timepoint. **Table S2a** Table of full algorithms. **Table S2b** Longworth Response mapping model. **Table S3** Performance of mapping algorithms in detecting clinical changes. Additional file 2:
**Distribution of EQ-5D values.** **A**- Observed values (UK tariff), **B**– Longworth algorithm, **C**– McKenzie algorithm.

## Abbreviations

EORTC: European organisation for research and treatment of cancer
MPM: Malignant pleural mesothelioma
NICE: National Institute for Health and Care Excellence
QALY: Quality adjusted life year
HRQoL: Health related quality of life
SF-6D: Short form- 6D
HUI: Health utilities index
RMSE: Root mean squared error
MAE: Mean absolute error
