# Supplementary material for: Testing mapping algorithms of the cancer-specific EORTC QLQ-C30 onto EQ-5D in malignant mesothelioma
Source: Health Qual Life Outcomes. 2015 Jan 23;13:6. doi: 10.1186/s12955-014-0196-y (PMC4316600; doi:10.1186/s12955-014-0196-y)
Supplement: Additional file 1: Table S1. — Table of missing questionnaires by timepoint. Table S2a Table of full algorithms. Table S2b Longworth Response mapping model. Table S3 Performance of mapping algorithms in detecting clinical changes. [file 12955_2014_196_MOESM1_ESM.pdf]

APPENDIX- Tables.

Supplementary Table 1: Table of missing questionnaires by timepoint.

	Total (n= 73)	Reasons questionnaires not received
Visit 1- Baseline		
Questionnaires completed (%)	72 (98.6)	Patient too unwell- 1
Visit 2- 6 weeks		
Questionnaires completed (%)	60 (82.2)	Patient too unwell-4<br>Chemo delayed- 9
Visit 3- 16 weeks		
Questionnaires completed (%)	54 (80.6)	Patient death- 6<br>Patient too unwell- 6<br>Withdrew from study- 2<br>Questionnaire incomplete- 5
Visit 4- 9 months		
Questionnaires completed (%)	33 (57.9)	Patient death- 22<br>Patient too unwell- 12<br>Withdrew from study-2<br>Questionnaire incomplete-4
Visit 5- 12 months		
Questionnaires completed (%)	31 (42.4)	Patient death- 32<br>Patient too unwell- 5<br>Withdrew from study-2<br>Questionnaire incomplete-3

Supplementary Table 2a; Table of full algorithms.

Mapping algorithm primary author	Algorithm
Crott	0.8592777 - (0.0069693 * PF) - (0.0087346 * EF) - (0.0039935 * SF) + (0.0000355 * PF^2) + (0.0000552 * EF^2) + (0.0000290 * SF^2) + (0.0011453 * CO) + (0.0039889 * DI) + (0.0035614 * PA) - (0.0003678 * SL) - (0.0000540 * DI^2) + (0.0000117 * SL^2)
Jang	0.3381 + (PF * 0.0035) + (RF * 0.0007) + (EF * 0.0011) + (CF * 0.0007) - (SF * 0.0007) + (QL * 0.0009) + (FA * 0.0003) - (NV * 0.0002) - (PA * 0.0021) - (DY * 0.0001) - (SL * 0.0001) - (AP * 0.0001) + (CO * 0.0005) + (DI * 0.0004) - (FI * 0.0001)
Kim EJ	0.5849 + (QL * 0.001) + (0.0035 * PF) - (0.0014 * PA) + (0.0008 * DY) - (0.0006 * AP)
Kim SH	0.56317 + (QL * 0.00097) + (0.00222 * PF) + (0.00067 * RF) + (0.00045 * EF) - (0.00125 * PA)
Kontodimopoulos	(PF2 * 0.00508) + (EF * 0.00313) + (QL2 * 0.00546) - 0.18143
Longworth	See table below for full model for calculating the probability of being in each response level. EQ-5D was then calculated by multiplying the probability of being in each response level by the standard UK tariff; 1−(Prmob2×0.069)−(Prmob3×0.314)−(Prcare2×0.104)−(Prcare3×0.214)−(Pruact2×0.036)−(Pruact3×0.094)−(Prpain2×0.123)−(Prpain3×0.386)−(Pranx2×0.071)−(Pranx3×0.236)−(1−PrPerfect)×0.081−PrN3×0.269
McKenzie	(QL * 0.0016) + (PF * 0.0004) + (RF * 0.0022) + (EF * 0.0028) + (CF * 0.0009) + (SF * 0.0002) - (FA * 0.0021) - (NV * 0.0005) - (PA * 0.0024) + (DY * 0.0004) + (SL * 0.00004) + (AP * 0.0003) + (CO * 0.0001) - (DI * 0.0003) - (FI * 0.0006) + 0.2376
Proskorovsky	0.25763 + (QL * 0.00165) + (PF * 0.00467) – (PA * 0.00293) + (SL * 0.0616)
Versteegh	0.978 - (q1 * 0.03) - (q2 * 0.025) - (0.045 * q3) - (0.069 * q4) - (0.159 * q5) - (q6_q2 * 0.037) - (q6_q3 * 0.077) - (q6_q4 * 0.187) - (q7_q3_q4 * 0.02) - (q9_q2_q3 * 0.076) - (q9_q4 * 0.267) - (q23_q2 * 0.02) - (q23_q3 * 0.028) - (q23_q4 * 0.267) - (q24_q2 * 0.071) - (q24_q3_q4 * 0.144) - (q27_q3 * 0.041) - (q27_q4 * 0.063)

QL- Global Health, PF- Physical Functioning, RF- Role Functioning, EF- Emotional Functioning, CF- Cognitive Functioning, SF- Social Function, FA- Fatigue, NV- Nausea/Vomiting, PA- Pain, DY- Dyspnoea, SL- Insomnia, AP- Appetite, CO- Constipation, DI- Diarrhoea, FI- Financial Difficulties.

Supplementary Table 2b; Longworth Response mapping model

Variables	Mob 2	Mob 3	SC 2	SC 3	UA 2	UA 3	Pain 2	Pain 3	A/D 2	A/D 3
PF	–0.072	–0.167	–0.049	–0.099	–0.036	–0.085	–0.001	–0.013	–0.014	–0.044
RF	–0.011	–0.007	–0.017	–0.030	–0.032	–0.055	0.001	–0.001	0.005	0.019
EF	0.010	0.024	0.008	0.008	0.021	0.028	0.009	0.011	–0.078	–0.148
CF	–0.011	–0.006	–0.010	–0.009	0.004	–0.001	0.003	0.015	–0.007	0.006
SF	0.003	0.011	–0.009	–0.005	–0.021	–0.034	0.005	–0.001	0.006	0.008
FA	0.006	0.002	–0.022	–0.025	0.028	0.033	0.007	0.006	–0.006	0.007
NV	0.001	0.016	0.007	0.019	0.022	0.022	0.005	–0.004	–0.007	–0.009
PA	0.023	0.043	0.016	0.024	0.020	0.023	0.100	0.164	0.002	–0.012
DY	0.002	0.004	–0.005	–0.015	–0.005	–0.015	0.010	0.008	0.000	–0.018
SL	0.002	0.010	0.002	–0.000	–0.001	–0.002	0.013	0.021	–0.003	0.012
AP	–0.009	0.004	–0.000	0.010	–0.010	–0.011	–0.013*	–0.008	0.006	0.016
CO	–0.004	–0.012	–0.004	–0.009	–0.000	0.004	0.006	0.010	0.004	0.001
DI	–0.005	0.010	0.003	0.005	–0.009	–0.011	–0.004	–0.008	0.002	0.002
FI	–0.001	–0.003	0.005	0.015	0.008	0.006	0.010	0.012	0.012	0.015
Age	0.028	–0.021	0.048	0.131					0.026	0.008
Female	–0.349	–1.397								
Constant	3.169	3.542	0.498	–6.619	3.494	5.675	–3.255	–9.819	4.562	

Mob- Mobility, SC- Self-care, UA- Usual activities, A/D- Anxiety/Depression.

Supplementary Table 3; Performance of mapping algorithms in detecting clinical changes.

Mapping algorithm used.	Chemotherapy (n= 41) vs Controls (n= 12)			Epithelial (n= 31) vs Sarcomatoid (n= 11)			Falling (n= 18) vs Rising mesothelin (n= 22)		
	Baseline EQ-5D	16 week EQ-5D	Same result found?	Baseline EQ-5D	16 week EQ-5D	Same result found?	Baseline EQ-5D	16 week EQ-5D	Same result found?
Observed EQ-5D	0.687<br>0.710	0.695<br>0.519	-	0.702<br>0.711	0.711<br>0.498	-	0.664<br>0.719	0.749<br>0.590	-
Crott	0.762<br>0.727	0.725<br>0.824	✗	0.771<br>0.721	0.768<br>0.690	✓	0.758<br>0.746	0.754<br>0.746	✗
Jang	0.756<br>0.741	0.714<br>0.782	✗	0.766<br>0.726	0.748<br>0.682	✓	0.774<br>0.729	0.742<br>0.734	✗
Kim EJ	0.891<br>0.897	0.848<br>0.903	✗	0.896<br>0.884	0.873<br>0.828	✓	0.905<br>0.883	0.852<br>0.875	✗
Kim SH	0.837<br>0.833	0.805<br>0.850	✗	0.841<br>0.826	0.831<br>0.778	✓	0.851<br>0.823	0.819<br>0.824	✗
Kontodimopoulos	0.788<br>0.805	0.712<br>0.802	✗	0.796<br>0.783	0.760<br>0.664	✓	0.806<br>0.763	0.724<br>0.759	✗
Longworth	0.688<br>0.738	0.693<br>0.636	✓	0.714<br>0.663	0.711<br>0.595	✓*	0.705<br>0.697	0.734<br>0.642	✓*
McKenzie	0.710<br>0.666	0.647<br>0.712	✗	0.722<br>0.656	0.702<br>0.574	✓*	0.711<br>0.667	0.674<br>0.661	✗
Proskorovsky	0.657<br>0.698	0.620<br>0.693	✗	0.672<br>0.651	0.639<br>0.549	✓	0.6548<br>0.6803	0.6535<br>0.6859	✗
Versteegh	0.838<br>0.824	0.798<br>0.815	✗	0.841<br>0.824	0.754<br>0.779	✓	0.852<br>0.822	0.693<br>0.763	✗

* Significant at p<·05 (ANCOVA)
