# Supplementary material for: Testing mapping algorithms of the cancer-specific EORTC QLQ-C30 onto EQ-5D in malignant mesothelioma
Source: Health Qual Life Outcomes. 2015 Jan 23;13:6. doi: 10.1186/s12955-014-0196-y (PMC4316600; doi:10.1186/s12955-014-0196-y)

Appendix 1. Distribution of EQ-5D values. A- Observed values (UK tariff), B– Longworth algorithm, C– McKenzie algorithm.

A)

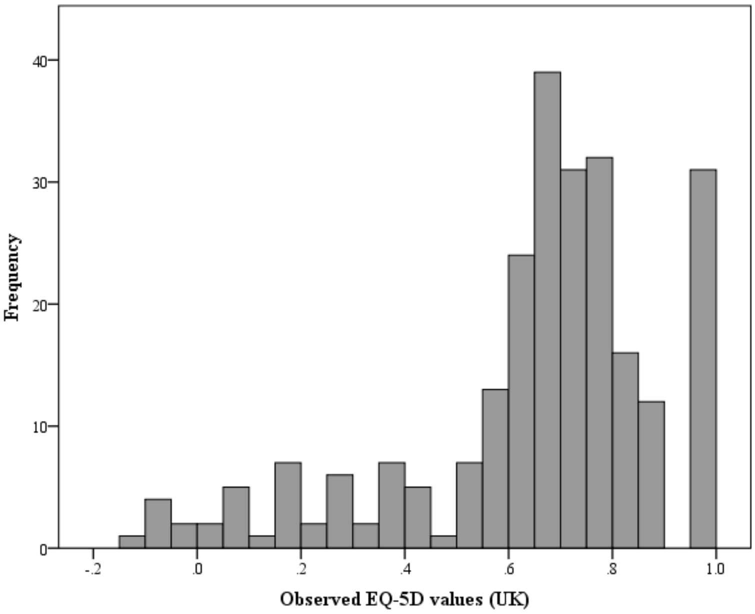

B)

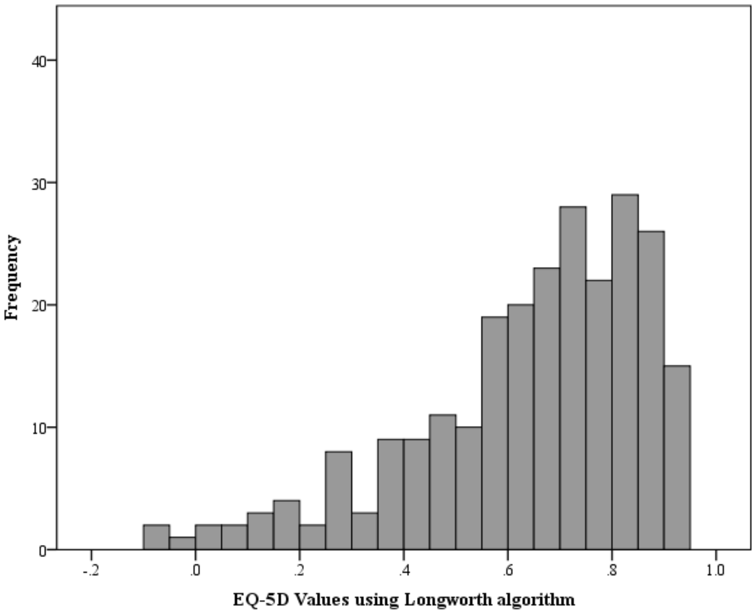

C)

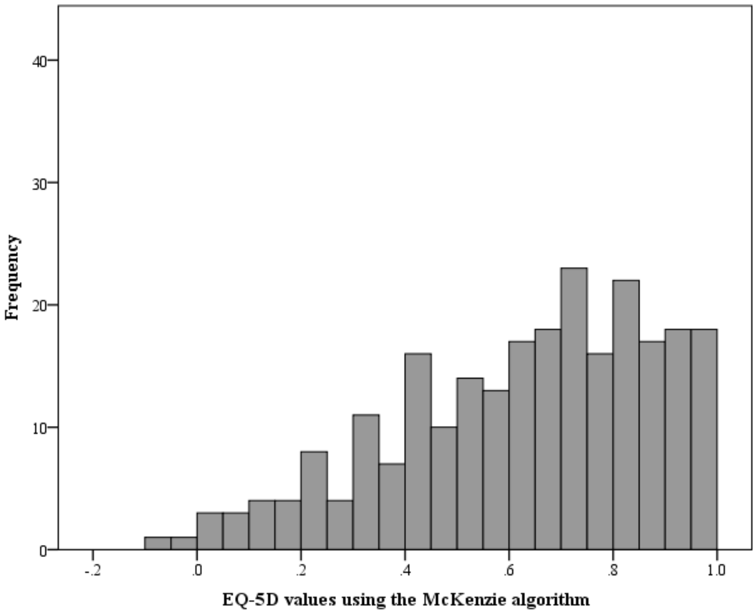

Supplement: Additional file 2: — Distribution of EQ-5D values. A- Observed values (UK tariff), B– Longworth algorithm, C– McKenzie algorithm. [file 12955_2014_196_MOESM2_ESM.pdf]
